# Progranulin modulates zebrafish motoneuron development *in vivo *and rescues truncation defects associated with knockdown of Survival motor neuron 1

**DOI:** 10.1186/1750-1326-5-41

**Published:** 2010-10-14

**Authors:** Babykumari P Chitramuthu, David C Baranowski, Denis G Kay, Andrew Bateman, Hugh PJ Bennett

**Affiliations:** 1 Endocrine Research Laboratory and Department of Medicine, Royal Victoria Hospital and McGill University Health Centre Research Institute, Montreal, Quebec, H3A 1A1, Canada; 2 Neurodyn Inc., Suite 508, NRC-INH, 550 University Avenue, Charlottetown, Prince Edward Island, C1A 4P3, Canada

## Abstract

**Background:**

Progranulin (PGRN) encoded by the *GRN *gene, is a secreted glycoprotein growth factor that has been implicated in many physiological and pathophysiological processes. PGRN haploinsufficiency caused by autosomal dominant mutations within the *GRN *gene leads to progressive neuronal atrophy in the form of frontotemporal lobar degeneration (FTLD). This form of the disease is associated with neuronal inclusions that bear the ubiquitinated TAR DNA Binding Protein-43 (TDP-43) molecular signature (FTLD-U). The neurotrophic properties of PGRN *in vitro *have recently been reported but the role of PGRN in neurons is not well understood. Here we document the neuronal expression and functions of PGRN in spinal cord motoneuron (MN) maturation and branching *in vivo *using zebrafish, a well established model of vertebrate embryonic development.

**Results:**

Whole-mount *in situ *hybridization and immunohistochemical analyses of zebrafish embryos revealed that zfPGRN-A is expressed within the peripheral and central nervous systems including the caudal primary (CaP) MNs within the spinal cord. Knockdown of zfPGRN-A mRNA translation mediated by antisense morpholino oligonucleotides disrupted normal CaP MN development resulting in both truncated MNs and inappropriate early branching. Ectopic over-expression of zfPGRN-A mRNA resulted in increased MN branching and rescued the truncation defects brought about by knockdown of zfPGRN-A expression. The ability of PGRN to interact with established MN developmental pathways was tested. PGRN over-expression was found to reverse the truncation defect resulting from knockdown of Survival of motor neuron 1 (smn1). This is involved in small ribonucleoprotein biogenesis RNA processing, mutations of which cause Spinal Muscular Atrophy (SMA) in humans. It did not reverse the MN defects caused by interfering with the neuronal guidance pathway by knockdown of expression of NRP-1, a semaphorin co-receptor.

**Conclusions:**

Expression of PGRN within MNs and the observed phenotypes resulting from mRNA knockdown and over-expression are consistent with a role in the regulation of spinal cord MN development and branching. This study presents the first *in vivo *demonstration of the neurotrophic properties of PGRN and suggests possible future therapeutic applications in the treatment of neurodegenerative diseases.

## Background

Progranulin (PGRN) (also called granulin-epithelin precursor, PC-derived growth factor, transforming growth factor-e, acrogranin or proepithelin) is a secreted glycoprotein that contains multiple linear repeats of the disulfide-rich granulin motif [1]. PGRN is implicated in numerous physiological and disease processes [2] including early development of the embryo [3-6], wound healing [7], inflammation [8,9], chondrogenesis [10] and cancer (reviewed in [2]). While it has long been known that PGRN is expressed in neurons of the adult and developing murine nervous system [11], roles for PGRN in the CNS have only recently emerged. *GRN *mutations cause a progressive neurodegenerative disease, namely frontotemporal lobar degeneration associated with ubiquitin inclusions (FTLD-U). FTLD-U belongs to the subset of neurodegenerative diseases that are characterized histologically by ubiquitin-positive cytoplasmic and intranuclear neuronal inclusions containing the TAR DNA binding protein, TDP-43 [12,13]. Familial FTLD is heterogeneous and in addition to *GRN *mutations, also arise from mutations of *MAPT*, the gene encoding the Tau protein. However, this variant differs histologically from FTLD-U in displaying Tau aggregation but lacking ubiquitinated TDP-43 inclusions [14]. To date 68 pathogenic autosomal dominant mutations have been reported to occur in the *GRN *gene [15]. The majority are nonsense mutations that lead to the introduction of premature stop codons resulting in elimination of the nascent PGRN mRNA through the process of nonsense mediated decay [14,16]. FTLD-U usually manifests initially either as behavioral or linguistic defects [17-19], and while it is predominantly an early onset form of dementia, the age of inception is highly variable ranging from 35-85 years [20]. This highly variable age-dependent penetrance suggests that the actions of PGRN are influenced by other gene products and possibly environmental factors. FTLD-U is invariably fatal and no effective treatment exists at present [21].

Many neurodegenerative conditions are associated with the accumulation of toxic protein aggregates including amyloid, tau-fibrils, synuclein and huntingtin [22,23]. The role of PGRN in FTLD-U is different in that the primary cause of the disease is partial loss of PGRN protein levels [19]. This results ultimately in neuronal cell death although the details of how this occurs are not well understood. While *Grn*-deficient mice do not appear to develop overt FTLD, recent studies of knockout mice emphasize how vulnerable neuronal tissue is to low PGRN levels. The sensitivity of hippocampal neurons to hypoxia-induced cell death is increased [24] and they exhibit enhanced aggressiveness and anxiety consistent with alterations in brain development [25]. Furthermore, PGRN has been implicated in the male-specific differentiation of the murine hypothalamus [26]. PGRN deficient mice display an exaggerated inflammatory response upon exposure to *Listeria monocytogenes *and chronic infection over time leads to greater activation of microglia and astrocytes and resultant neuronal damage [24]. Progranulin deficient mice display a range of behavioural deficits and progressive neuropathological defects [27]. Knockout mice also display increased neuronal lipofuscinosis and ubiquitination as they age suggesting a critical role for PGRN as a long-term neuronal survival factor [28]. We, and others, have shown that PGRN is neuroprotective *in vitro *[29,30], but little is currently known about how PGRN affects neuronal survival and development *in vivo*. This is clearly an important question given the devastating clinical effects of partial loss of PGRN expression in FTLD patients. Here we employed the zebrafish animal model to investigate this issue.

The zebrafish (*Danio rerio*) has emerged as a powerful tool to study early developmental events, to define gene function and to model human disease [31]. Unlike the mammalian genome that contains only a single *GRN *gene, the zebrafish possesses four distinct *GRN *genes. Two of these genes, namely *grna *and *grnb *resemble mammalian *GRN *in possessing multiple tandem repeats of the GRN motif. Syntenic conservation of gene localization suggests that of these, *grna *is the true orthologue of the human *GRN *gene [32,33] and was therefore the major focus of this study. In previous studies we have identified central and spinal cord motoneurons as among the most highly PGRN-expressing cells in the murine nervous system and demonstrated a cytoprotective action for PGRN on MN-like cells *in vitro *[30]. This prompted an examination of PGRN in MN development in zebrafish.

During the first 24 hours post-fertilisation (hpf) in zebrafish primary caudal (CaP), mid (MiP), and rostral (RoP) motor neurons initially share a common pathway. This is the developmental period encompassed in our studies. Normal CaP neurons rarely branch before the horizontal myoseptum (HM) [34], an assembly of muscle pioneer cells which serves as a transient target for developing primary motoneurons prior to the development of axonal projections that extend toward target populations of muscles cells. The CaP neurons develop first and extend ventrally beyond the horizontal myoseptum where branching promotes functional contact within the myotome. The development of CaP neurons is a well characterized experimental model to study mechanisms that regulate MN axonal development and projection [34]. Axonal projection and branching are readily visualized in embryo whole-mounts and antisense mRNA knockdown of factors that specifically disrupt CaP development have been reported [35,36]. Growth cone guidance is provided by secreted factors including semaphorins, netrins and ephrins [37]. MN differentiation and survival is also influenced by morphogens and other humoral factors including IGF [38], Wnt and Shh signaling [39]. Our results suggest that PGRN may also play a role in regulating these processes.

## Results

### Zebrafish progranulin-A is expressed within the Central Nervous System (CNS) and Peripheral Nervous System (PNS)

**Figure 1:**
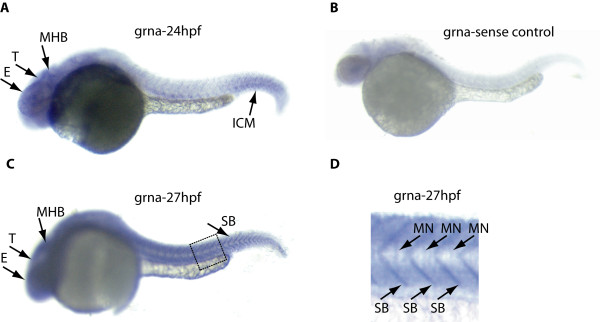
**Zebrafish PGRN-A *in situ *expression in the Spinal cord and MNs**. Lateral view of 24-27 hpf embryos revealed zfPGRN-A expression within the Peripheral and Central Nervous systems. In the head region, zfPGRN-A is expressed at 24 hpf in the head and brain particularly in the tectum, midbrain-hindbrain boundary and eye (Panel A). The sense control displayed minimal signal (Panel B). In addition zfPGRN-A is also expressed at 27 hpf in the somite boundaries (Panel C). At greater magnification of the boxed area in Panel C, zfPGRN-A expression in the somite boundaries is more apparent together with expression in the motoneurons (Panel D). Abbreviations: T: Tectum; E:Eye, MHB:Mid-Brain, Hind-brain Boundary; SB:Somite Boundary, MN:Motoneurons; ICM: Intermediate cell mass. Images were captured at 6X magnification and the hatched box was further subject to 2-3X Zoom

**Figure 2:**
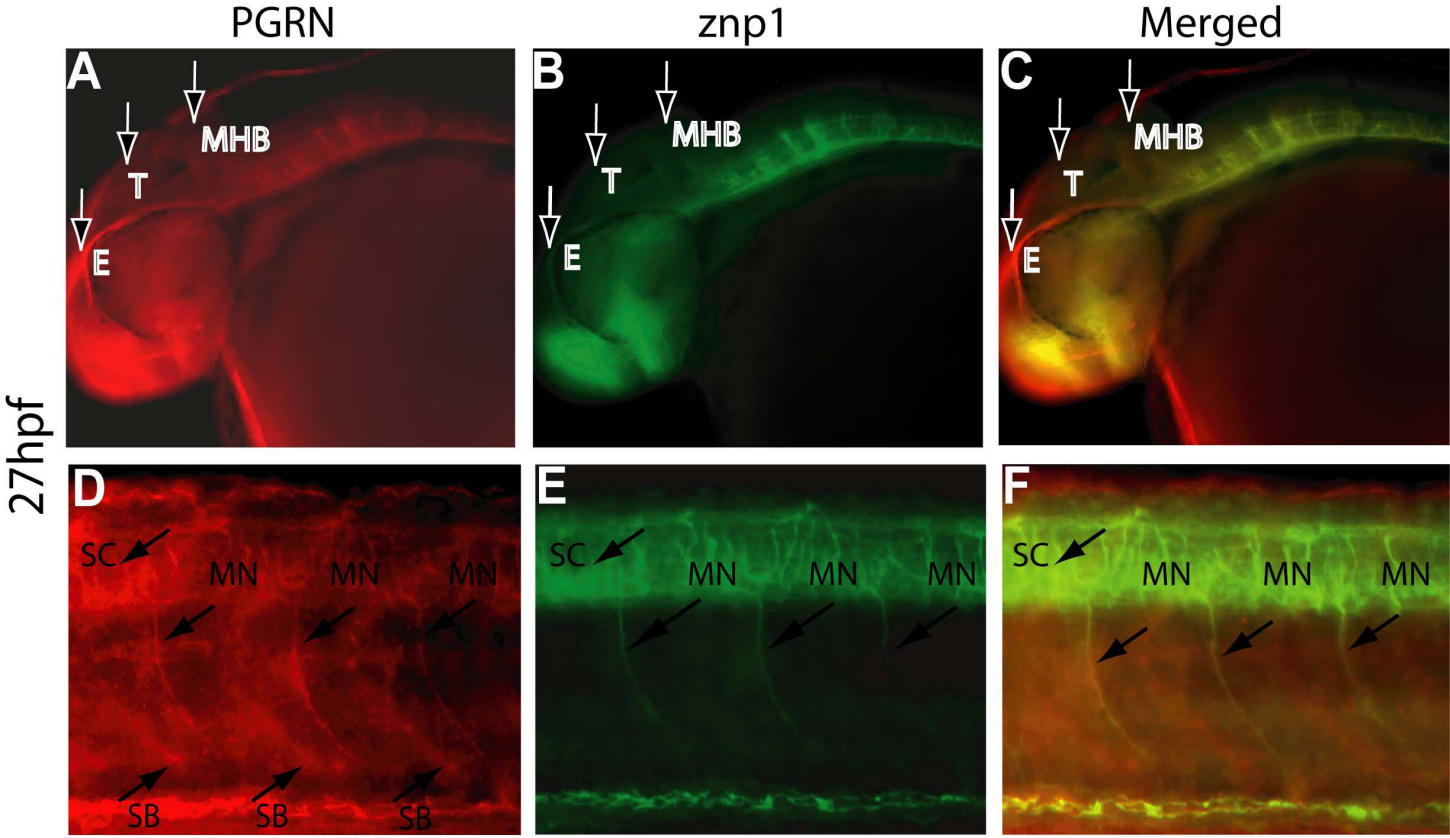
**Colocalization of zfPGRN-A and znp1 immunoreactivity in Primary motor neurons at 27 hpf**. Lateral views (anterior- left; dorsal- top) of whole-mount embryos labelled with zfPGRN-A (A, D) and znp1 mAb (B, E) at 27 hpf. Embryos show zfPGRN-A expression in the developing eye, tectum and hindbrain (A); Spinal Cord, Somite boundaries and motor neurons (D); znp1 expression in the eye, hindbrain, Spinal Cord and motor neurons (B and E). Merged images show co-localization of zfPGRN-A and znp1 (C, F). Abbreviations: T, Tectum; E, Eye; MHB, Mid-Brain Hindbrain Boundary; SB, Somite boundary; MN, Motoneurons.; SC, Spinal cord. Images were captured at 20 × magnification. Scale Bar = 50 μm.

In our initial studies of zebrafish PGRN (zfPGRN) expression [32] we demonstrated that there is general neural expression of zfPGRN-A in the hindbrain and tectum during the first 24 hpf. Given the high expression of PGRN in murine MNs we investigated zfPGRN-A expression using whole mount *in situ *hybridization at 27 hpf with specific emphasis on expression within MNs (Figure 1, Panels A and C). Expression of zfPGRN-A was detected during neural development in the retina, hindbrain, tectum, spinal cord and caudal primary (CaP) motoneuron (MNs). At 27 hpf zfPGRN-A expression was also detected in the somite boundaries, a potential source of factors influencing MN development (Figure 1, Panel C and D). A similar tissue-distribution pattern was observed for immunoreactive zfPGRN-A using whole mount immunofluorescence (Figure 2, Panels A, D, C and F). Control experiments where the primary antibody is replaced with normal calf serum to eliminate the possibility of non-specific staining did not produce an immunoreactive signal (data not shown). The expression of zfPGRN-A within the MNs was further confirmed by the co-localization with znp1, a marker for MNs (Figure 2, Panels B, C, E and F). These observations are consistent with murine neural expression of PGRN during development [11].

### zfPGRN-A knockdown results in disrupted development of multiple neural structures

**Figure 3:**
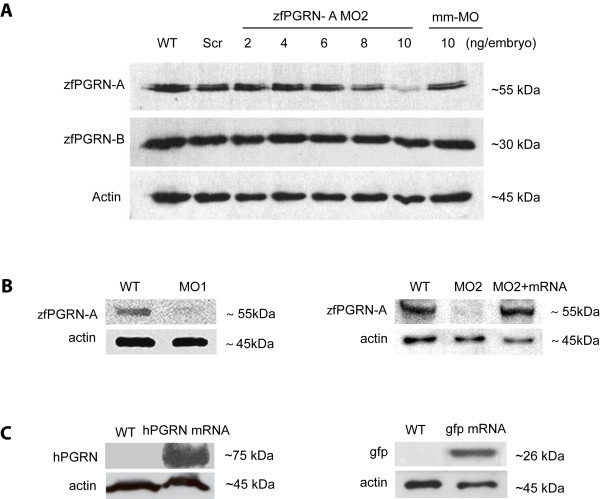
**zfPGRN-A knockdown, rescue and over-expression**. (A) Dose-dependent efficacy of morpholino (MO2) targeting the 5' UTR of zfPGRN-A was assessed by Western blot analysis in comparison to its co-orthologue zfPGRN-B and actin. zfPGRN-A and B are represented by single bands of approximately 55 and 30 kDa, respectively. The Western blot analysis of MO knockdown efficacy is a representative of three independent injection sets. (B) Western blot analysis of protein extracts from embryos injected with 10 ng of zfPGRN-A MO1, 10 ng of zfPGRN-A MO2, zfPGRN-A-MO2 together with zfPGRN-A mRNA. Injection of 10 ng/embryo of the zfPGRN-A MO1 and MO2 effectively decreased zfPGRN-A translation, zfPGRN-A-MO together with mRNA resulted in normal zfPGRN-A translation. (C) Control experiments showing the results of the injection of 100pg hPGRN mRNA and 1ng gfp mRNA demonstrating translation into protein for both.

**Figure 4:**
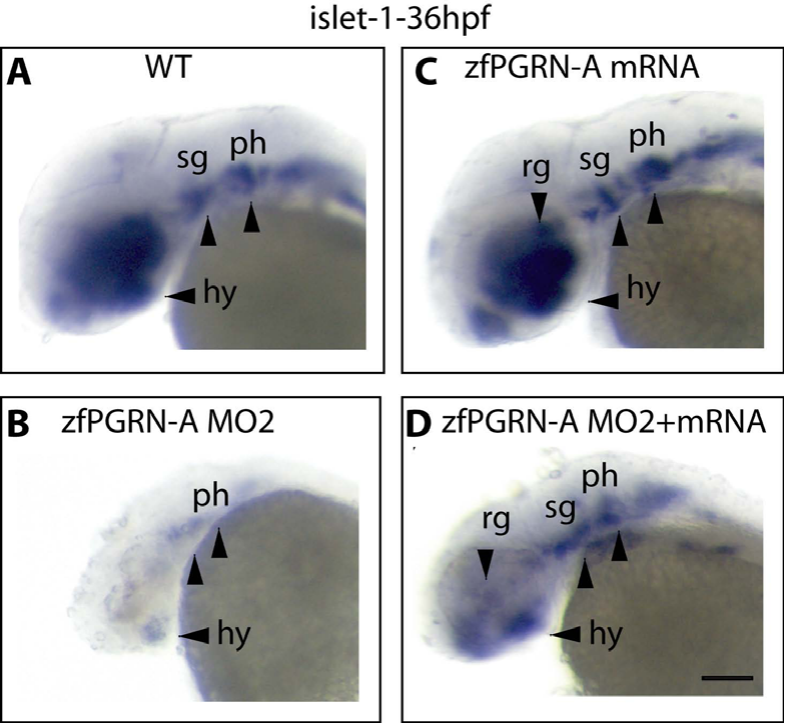
**zfPGRN-A knockdown results in disrupted development of multiple neural structures**. Lateral views of whole-mount embryos probed with islet 1 at 36 hpf. (A), uninjected embryos (B) embryos injected with zfPGRN-A MO2 (C) embryos injected with 100 pg zfPGRN-A mRNA, or (D) embryos co-injected with zfPGRN-A MO2 + 100 pg zfPGRN-A. 96% (48/50) of uninjected embryos (A) and 90% (45/50) of embryos injected with 100 pg zfPGRN-A mRNA (B) showed islet1 expression in the developing hypothalamus, retinal ganglia, sensary ganglia and pharyngeal arches. 84%(31/37) of embryos injected with 10 ng of zfPGRN-A MO2 (C) showed disrupted development of multiple neural structures. Co-injection of embryos with 10 ng MO2 and 100 pg zfPGRN-A mRNA (D) resulted in the rescue of 80% (40/50) embryos displaying islet 1 positive neural populations. Scale Bar = 100 μm

Antisense morpholino (MO) technology was employed to investigate the function of zfPGRN-A in neural development by knocking down gene expression by injection oligonucleotides into zebrafish embryos at the 1 to 2 cell stage. Two independent zfPGRN-A MO were employed to inhibit zfPGRN-A translation directed either against the zfPGRN-A ATG (designated as MO1) or the 5'UTR (designated as MO2). Titration of the concentration of injected zfPGRN-a MO2 against zfPGRN-A levels demonstrated that 10 ng/embryo of the zfPGRN-A MO effectively decreased zfPGRN-A translation (Figure 3, Panel A) without affecting the expression of the orthologue zfPGRN-B. Injection of the equivalent amount of a 5 base-pair mismatch control morpholino (mm) or an equivalent scrambled sequence (scr) resulted in little or no decrease in zfPGRN-A expression (Figure 3, Panel A). Co-injection of zfPGRN-A-MO with zfPGRN-A mRNA restored zfPGRN-A protein levels (Figure 3, Panel B). The efficacy of translation from injected mRNA was confirmed by injection of hPGRN mRNA or control green fluorescent protein (gfp) mRNA which were shown by Western blot to be translated into protein products (Figure 3, Panel C). GFP translation was observed throughout the embryo (Additional File 1). Embryonic injection of 10 ng zfPGRN-A MO resulted in disrupted development of multiple neural structures at 36 hpf (assessed by islet-1 expression) that was partially rescued by co-injection with zfPGRN-A mRNA (Figure 4). The expression pattern, knockdown and rescue experiments suggest roles for PGRN in CNS development.

### Knockdown of PGRN-A expression generates defects in motoneuron development

**Figure 5:**
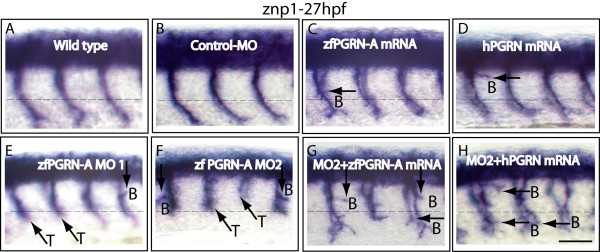
**Progranulin modulates motoneuron development *in vivo***: zfPGRN-A knockdown induced CaP MN defects that were partially rescued by either zfPGRN-A or hPGRN mRNA. Lateral views (anterior to the left; dorsal to the top) of embryos labelled with znp1 mAb at 27 hpf in (A) wild type embryos, (B) embryos injected with Control MO, (C) 100 pg zfPGRN-A mRNA, (D) 100 pg hPGRN mRNA, (E) zfPGRN-A MO1, (F) zfPGRN-A MO2, (G) zfPGRN-A MO2+100 pg zfPGRN-A mRNA and (H) zfPGRN-A MO2+100 pg hPGRN mRNA. Observed phenotypes were normal MN development (A, B), increase in branched axons (C-H), truncation of axons (E, F) and partial rescue of truncated MNs (G, H). Dashed lines represent horizontal myoseptum. Abbreviations: T-truncation; B-Branching. Scale Bar = 50 μm.

**Figure 6:**
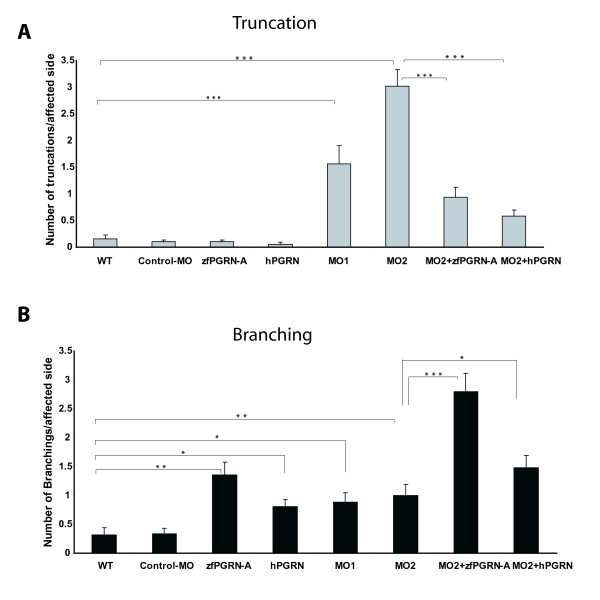
**Progranulin knockdown and over-expression validation**: Average number of Truncated (A) and Branched (B) CaP MNs (n≥50/group). Values were expressed as mean ± standard error of the mean. Statistical analyses were done with one-way ANOVA followed by student-Newman-keuls Multiple Comparisons Test (p < 0.001-***, p < 0.01-**, p < 0.05-*). Error bars represent s.e.m. Abbreviations: WT, wild type; Control-MO, Control Morpholino; zfPGRN-A, zebrafish PGRN-A mRNA; hPGRN, human progranulin; MO1, Morpholino directed against the initiator codon ATG; MO2, Morpholino directed against the 5'UTR.

We visualized the development of the CaP neurons in whole mount embryos at 27 hpf by immunostaining using the znp1 monoclonal antibody that labels primary MNs (Figure 5). Data sets are presented as lateral images of developing primary MNs found within three or four somatic hemisegments of the 12-13 found in each developing embryo. This occurs on both sides of the body and accounts for the apparent image duplication seen with such images. This can be seen for example in several of the panels shown in Figure 5. CaP MNs were scored for truncation and aberrant branching. Both wild type and embryos injected with control morpholino (MO) show normal CaP MN development (Figure 5, Panels A and B; Figure 6). Knockdown of zfPGRN-A caused truncation and frequent premature branching of CaP MNs proximal to the HM at 27 hpf (Figure 5, Panels E and F). The incidence of both truncated and premature branching of CaP motoneurons generated by PGRN-A knockdown is significant when compared to embryos injected with control MO or wild type embryos (Figure 6). To evaluate whether reduction of zfPGRN-B expression disrupts the formation of neural structures and motoneuron development we knocked down zfPGRN-B using an MO directed against the UTR region of PGRN-B. The depletion of PGRN-B produced a severe phenotype notably including a reduction in head size seen at 24 hpf (data not shown). This is consistent with the high levels of mRNA provided maternally to the newly fertilised egg and the extensive expression of zfPGRN-B found throughout larval brain. These observations make it difficult to analyze the possible role of zfPGRN-B in motoneuron development at this time.

### PGRN-A stimulates motoneuron branching and rescues the truncation defects resulting from knockdown of PGRN-A expression

Ectopic expression of either zfPGRN-A mRNA or hPGRN mRNA increased the incidence of branching (Figure 5, Panels C and D; Figure 6) without causing truncation defects. Co-injection of either zfPGRN-A mRNA or hPGRN mRNA together with zfPGRN-A MO1 or 2 significantly reduced the incidence of truncated neurons suggesting that zfPGRN-A is necessary for normal CaP MN development (Figure 5, Panels G and H; Figure 6, Panel A). In addition, the co-injection of either zfPGRN-A mRNA or hPGRN mRNA with zfPGRN-A MO 1 or 2 increased the incidence of early and late branching when compared to MO injection. These studies suggest that zfPGRN-A promotes neuronal outgrowth *in vivo *(Figure 5, Panels C, D, G and H; Figure 6, Panel B). The phenotypes were validated in each category with 3 independent experiments with sample sizes of at least 20 (WT, Control, PGRN MO1, PGRN MO2, PGRN MO2 plus PGRN-A mRNA and PGRN MO2 plus hPGRN mRNA).

### Knockdown of zfPGRN-A impedes the avoidance swim response

To determine whether the motoneuron defects observed when zfPGRN-A expression was depleted were manifest as functional defects we tested the embryo touch response and avoidance swimming behaviour 48 hrs post injection. While the touch response is unaltered, PGRN-A deficient embryos show a marked swimming defect. Embryos do not show the strong avoidance swimming phenotype typical of wild-type embryos. At 72 hrs post injection the motility of zfPGRN-A deficient embryos is further reduced and that this defect worsens progressively beyond 48 hpf. This suggests that while there is no major sensory deficit there is a motor deficit possibly due to defective innervation of MNs in the PGRN-A knockdown embryos (Additional File 2, Supplementary Table S1; Additional Files 3, 4, 5, 6, Supplementary Videos 1-4;). The defect was rescued by co-injection of either zfPGRN-A or hPGRN-A together with zfPGRN-A MO1 (Additional File 2, Supplementary Table S1; Additional Files 7 and 8, Supplementary Videos 5 and 6). The majority of the embryos over-expressing either zfPGRN-A or hPGRN-A show the strong avoidance swimming phenotype typical of wild-type embryos (Additional File 2, Supplementary Table S1; Additional Files 9 and 10, Supplementary Videos 7 and 8).

### zfPGRN-A reverses the smn1 knockdown phenotype *in vivo*

**Figure 7:**
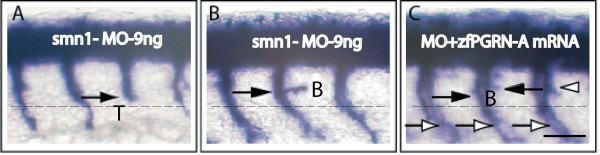
**Progranulin protects CaP MNs in a model of Spinal Muscular Atrophy SMA)**: zfPGRN-A mRNA co-injection partially rescues SMN-MO induced CaP MN defects. (A, B) Lateral views of embryos injected with 9 ng SMN-MO and (C) embryos co-injected with 9 ng SMN-MO + 100 pg zfPGRN-A mRNA. Symbols as in Figure 1. Truncated (A) and branched (B) neurons were observed upon injection of SMN MO while co-injection with zfPGRN-A rescued truncation (white arrow) and increased axon branching (C). Abbreviations: T-truncation; B-Branching. Scale Bar = 50 μm.

**Figure 8:**
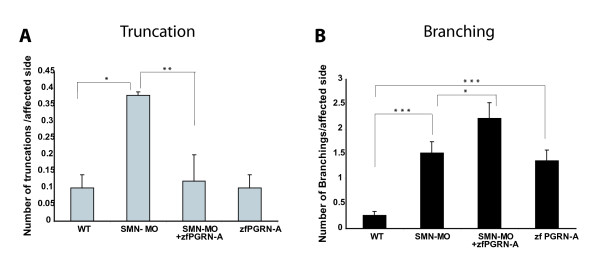
**Validation of rescue effect of zfPGRN-A in SMA model**: Average number of specified MN defects per affected side (n ≥ 50/group). Statistical analyses were performed with one-way ANOVA followed by student-Newman-keuls Multiple Comparisons Test (p < 0.001-***, p < 0.01-**, p < 0.05-*). Error bars represent s.e.m. Abbreviations: WT, wild type; zfPGRN-A, zebrafish PGRN-A mRNA; smn1-MO-co-injection of Morpholino directed against initiator ATG and Morpholino directed against 5'UTR.

**Figure 9:**
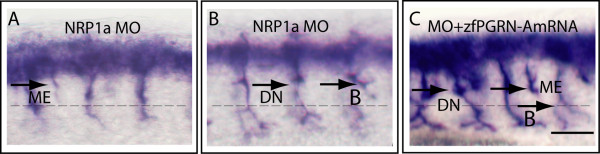
**Co-injection of zfPGRN-A mRNA exaggerated NRP1a-MO induced Cap MN defects particularly branching**. (A, B) Embryos injected with NRP1a-MO and (C) co-injected with NRP1a-MO + 100 pg zfPGRN-A. Multiple exits (A, C), Branched motor axons (B and C), and displaced neurons (B, C) were observed. Abbreviations: ME, Multiple Exits; DN, Displaced Neurons; B, Branching. Scale Bar = 50 μm.

To determine whether zfPGRN-A modulates the actions of factors known to modify MN developmental fate, we investigated the ability of zfPGRN-A to rescue defects in previously characterised motoneuron developmental pathways. Two such pathways were chosen; knockdown of Survival of Motorneuron-1 *SMN1*, a protein with important roles in spliceosomal small nuclear ribonucleoprotein biogenesis and axon outgrowth [40] and *Neuropilin 1a*, a semaphorin co-receptor that interacts with other mediators of the semaphorin pathway to regulate axonal guidance [41]. Mutations in human *SMN1 *are known to cause spinal muscular atrophy (SMA) [42], the most common genetic cause of early lethality in children and abnormal variants have been implicated in sporadic ALS [43]. MO-induced knockdown of SMN resulted in truncated and aberrantly branched CaP neurons that, as reported previously, reproduce aspects of human SMA [35], (Figure 7, Panels A and B). Co-injection of the smn1 MO with zfPGRN-A mRNA reversed the truncation defects. Branching increased relative to the results obtained with MO alone (Figure 7 Panel C). Phenotypes were validated using at least 50 embryos in each category and the data is presented as histograms in Figure 8, Panels A and B. To determine the rescue effect of PGRN in reversing motoneuron defects due to smn1 knockdown at a functional level we tested the embryo touch response and avoidance swimming behaviour 48 hrs post injection. While the smn1 knockdown embryos do not show the strong avoidance swimming phenotype typical of wild-type larvae, the embryos co-injected with either zfPGRN-A or hPGRN show an improved avoidance swimming phenotype when compared to that resulting from knockdown of smn1 (Additional File 2, Supplementary Table S1; Additional Files 11, 12, 13, Supplementary Videos 9-11). It is also interesting to note that about 85 percent of embryos co-injected with either zfPGRN-A or hPGRN together with smn1 MO survived 24 hrs post injection whereas the smn1 knockdown embryos showed only 50 percent survival 24 hrs post injection. These studies suggest that PGRN is protective against the MN truncation observed in a model of SMA and confirms that PGRN is a potent stimulator of neuronal branching *in vivo. *As reported previously [36] MO-induced knockdown of NRP-1a mRNA causes multiple exits, branching and displaced MNs but resulted in no truncation defects (Figure 9, Panels A and B). Co-injection of NRP1a MO with zfPGRN-A mRNA increased branching versus NRP-1a MO alone (Figure 9, Panel C) but did not prevent multiple exits or displaced neurons.

## Discussion

While it is known that PGRN is cytoprotective for neurons *in vitro *[29,30], and its partial loss results in neurodegeneration in FTLD-U [14,16] little is known how PGRN affects neurons *in vivo*. Here we employed zebrafish to investigate this important question. The zebrafish harbors an extended PGRN gene family with four distinct members [32]. Both *grna *and *grnb *encode precursors structurally homologous to the single human gene and are expressed within the CNS. High maternal expression of grnb was observed. In addition, grna is also expressed maternally but at much lower levels and this expression increases over time [32]. The other *GRN *genes *grn1 *and *grn2 *are expressed weakly at 48 hpf and only become strongly expressed in the intestine and pharyngeal region at 3 dpf and beyond [32]. We focused our study on *grna *because (1) it is true orthologue of the single human gene (2) it is expressed within the CNS and (3) the lower level of maternal expression may result in less severe phenotypes when expression is knocked down. We observed that the expression of grna occurs during neural development in the retina, hindbrain, tectum (Figure 1 and 2) and that depletion of zfPGRN-A results in a general disruption of neuronal development as assessed by the islet-1 marker during the first 24 hpf and beyond (Figure 4, and data not shown). Following our finding that mouse motor neurons express PGRN [30] we examined the expression of zfPGRN-A at both mRNA and protein levels to determine whether grna is expressed within zebrafish MNs. Both caudal primary (CaP) motoneurons (MNs) and somatic boundaries express zfPGRN-A during this period (Figure 1 and 2) confirming that the spatiotemporal distribution of zfPGRN-A is consistent with a role in the development of MNs. zfPGRN-A generated in the somatic boundary may provide a paracrine stimulus for MN development, while intrinsic MN expression of zfPGRN-A may serve an autocrine function. The extent to which either PGRN containing compartment, namely the MNs and the somatic boundary contribute to MN development and whether they serve distinct or overlapping functions in the developmental process remains unclear at present.

The developing zebrafish MN provides a very advantageous system to study the ontogeny of individual vertebrate neurons *in vivo *and in real time. Primary MNs initiate a common pathway shared by all three classes of primary motoneuron to a "choice point" where the growth cones pause when they contact a group of specialized cells called muscle pioneers at the horizontal myoseptum [34] whose location is indicated by a dashed horizontal line in the lateral views of zebrafish embryos shown in Figures 5, 7 and 9. After pausing, each growth cone, in turn, selects a different pathway that leads to a specific population of muscle cells. The CaP growth cones continue to grow in a ventral direction. Development of CaP neurons were examined in whole-mount embryos 26-28 hpf and visualized by immunostaining using the MN marker znp1. Experiments were designed to test the consequences of 1) zfPGRN-A over-expression 2) zfPGRN-A knockdown and 3) rescue of zfPGRN-A knockdown by zfPGRN-A mRNA. In addition the ability of human PGRN (hPGRN) mRNA to reproduce the actions of zfPGRN-A was tested. Rescue experiments involved the co-injection zfPGRN-A MO2 together with zfPGRN-A mRNA or hPGRN mRNA. Since the UTR sequence adjacent to the initiator AUG that is targeted by the MO is absent from the exogenous zfPGRN-A mRNA, translation of the injected mRNA is unaffected by the co-injected MO. To control for the appearance of developmental defects potentially resulting from the injection and expression of exogenous protein and the associated experimental manipulations, mRNA encoding GFP was injected into embryos at the 1-2 cell stage. The expression of GFP fluorescence was followed (Additional File 1). At 24 hpf no gross developmental changes were observed and importantly the GFP was widely expressed suggesting that all pertinent tissues and cells are impacted when undertaking mRNA over-expression and rescue experiments.

Knockdown of zfPGRN-A generated truncation of CaP neurons indicative of a role in the formation of MNs. Co-injection of zfPGRN-A or hPGRN mRNA with zfPGRN-A MO2 resulted in a partial rescue (Figures 5 and 6). Wild type development of CaP neurons shows branching only beyond the horizontal myoseptum, however over-expression of zfPGRN-A or hPGRN increased branching both before and beyond the horizontal myoseptum. Since ectopic expression of injected mRNA is ubiquitous (Additional File 1) this may reflect the loss of topographical specificity in PGRN stimulation of the developing MN. The disruption of MN structures caused by depleting zfPGRN-A translation is paralleled by changes in gross motility in which the touch response remains intact but touch-evoked swimming is greatly impaired (Additional File 2, Supplementary Table S1; Additional Files 3, 4, 5, 6, Supplementary Videos 1-4). The motility defect was partially rescued when the embryos were co-injected with either zfPGRN-A or hPGRN together with the zfPGRN-A MO. (Additional File 2, Supplementary Table S1, Additional Files 7 and 8, Supplementary Videos 5 and 6). These results imply (i) that PGRN is required for normal MN development (ii) that PGRN stimulates neuronal branching *in vivo *and that (iii) in this context zfPGRN and hPGRN are functionally equivalent. These results do not establish whether PGRN acts directly on the MNs or initiates a cascade of signals that regulate MN development indirectly. A definitive answer to this question will require a better understanding of the distribution and properties of PGRN receptors. However, over-expression of PGRN promotes neurite-like extensions in monocultures of MNs and MN cell lines under highly defined conditions and suggests a direct effect of PGRN upon the developing MN [29,30]

Interestingly, unlike the zebrafish, the knockout of *GRN *in mice does not result in marked MN defects [44]. This is perhaps surprising given the abundant expression of PGRN in murine MNs, its ability to regulate neurite extension and survival in murine MN cells in culture [29,30], the association of *GRN *mutations with amyotrophic lateral sclerosis as a putative susceptibility factor [45] and the results obtained in the zebrafish model reported in the present study. We suspect that this implies the existence of highly redundant survival mechanisms with respect to mammalian MNs which in comparison with the zebrafish counterparts are both exceptionally elongated and long-lived.

Since these results indicate a novel role of zfPGRN-A in spinal cord MN development and branching we investigated whether zfPGRN-A would interact with other factors that are known to regulate MN development. zfPGRN-A mRNA expression reversed the truncation defects brought about by smn1 knockdown *in vivo*. Intriguingly TDP-43 and SMN are both involved with RNA processing or stabilization. That PGRN is associated biologically with both TDP-43 [13] and smn1 (Figure 7, 8) may indicate shared pathways in their respective pathologies. It is noteworthy that the depletion of zfPGRN-A, snm1, as reported here and TDP-43 as reported elsewhere [46] results in a very similar MN phenotype namely truncation with excess branching. Knockdown of NRP-1a, a semaphorin co-receptor involved in neuronal pathfinding, resulted in multiple exits and misplaced MNs but not their truncation and this spectrum of defects was not reversible by co-expression of zfPGRN-A. The excessive branching seen following the depletion of smn1 or NRP-1a was significantly exaggerated in the presence of co-injected zfPGRN-A mRNA providing further evidence for the ability of zfPGRN-A to promote MN branching in the zebrafish CaP developmental model.

## Conclusions

PGRN regulates spinal cord MN development and branching. PGRN is expressed within the zebrafish CaP motoneuron during development. PGRN knockdown generates both truncated CaP MNs and premature branching while ectopic over-expression promotes branching. zfPGRN-A mRNA rescues truncation defects *in vivo. *Consistent with the emerging appreciation of the neuroprotective properties of PGRN [29,30], zfPGRN-A over-expression reverses the truncation effect of SMN knockdown, mutations of which cause SMA in humans. This study presents the first *in vivo *demonstration of the neurotrophic properties of PGRN and suggests possible future roles in the therapy of neurodegenerative conditions.

## Materials and methods

### Fish husbandry

Wild type zebrafish were purchased from Aquatica Tropicals (Florida) and maintained on a 14 h/10 h light/dark cycle at 28.5°C in a laboratory aquarium (Allentown Caging Equipment Co. Inc., Allentown, NJ). Zebrafish were maintained according to protocols approved by McGill University animal Care Committee (Protocol Number 3935). Fish were fed twice daily. In the late afternoon of the day before embryos are required (approximately 3:00 p.m.), fish were transferred to a net positioned towards the top of a holding tank and covered. In the morning, after the light cycle begins and spawning has stopped, the eggs that have fallen through the net were collected from the bottom of the tank. Embryos to be used for developmental studies were collected and staged by hours post fertilization (hpf) [47].

### Whole-mount zebrafish *In situ *hybridization

Analysis of gene expression by ISH was carried out essentially as previously described [32] using zfPGRN-A and Islet 1 sense and anti-sense DIG-labelled riboprobes. Stained whole-mount embryos were mounted in glycerol and visualized under a Leica MZFLIII stereomicroscope. Pictures were taken with a Leica DC350F camera and processed with Adobe Photoshop 7.0 software.

### Embryo microinjection of morpholino oligonucleotides

Morpholino oligonucleotides (MO) were obtained from Gene Tools, Inc. (Philomath, OR) and diluted in Danieaux buffer (58 mM NaCl, 0.7 mM KCl, 0.4 mM MgSO4, 0.6 mM Ca(NO3)_2_, 5.0 mM Hepes pH 7.6) containing 0.05% phenol red [48]. Approximately 2 nL of Morpholino along with 0.05% FITC-dextran (Sigma-Aldrich, Oakville, ON, Canada) was injected into the yolk of 1- to 2-cell stage embryos using a PLI-100 microinjection system (Harvard Apparatus, St. Laurent, QC, Canada). Phenotype observation and documentation were accomplished using a Leica DC300F digital camera connected to a Leica MZFLIII stereomicroscope and processed with Adobe Photoshop 7.0 software. The sequence of MO directed against the initiator AUG of zfPGRN-A (MO1) was 5'GAGGCAGACTGTCAGTCTCAACATT3' and the sequence of the MO corresponding to the 5'UTR region of zfPGRN-A (MO2) was 5'GAGCAGGTGGATTTGTGAACAGCGG3'. The sequence of the MO corresponding to the 5'UTR region of zfPGRN-B was 5' TACAGATGAAAAGCCATGAACGACT3'. MOs directed against the initiator AUG of smn1 (5'CGACATCTTCTGCACCATTGGC3'), the UTR sequences of smn1 (5'TTTAAATATTTCCCAAGTCCAACGT3') and NRP1a (5'GAATCCTGGAGTTCGGAGTGCGGAA3') were also used. For Morpholino injection 10 ng zfPGRN-A, 9 ng of smn1(both the MOs together) or 8 ng of NRP1a were used.

### Microinjection of PGRN mRNA

For zfPGRN-A mRNA over-expression and rescue experiments a full-length zfPGRN-A/pcDNA3 vector was generated as follows: The full-length zfPGRN-A sequence was purchased from RZPD (Berlin, Germany) as clone UCDMp574E2318Q2 and subcloned into pcDNA3.1-V5/His vector (Invitrogen, Carlsbad, CA) using a forward primer that overlapped with the starter AUG and a reverse primer that read through the termination codon. The final vector construct consisted of full-length zfPGRN-A with a carboxyl-terminal tag consisting of the V5 epitope and 6×Histidine. The authenticity of the construct was verified by DNA sequencing. Other vectors used were full-length hPGRN/pcDNA3^7^and GFP/pcDNA3 (Invirogen). For the zfPGRN-A rescue experiments the MO directed against the 5'UTR region of zfPGRN-A (MO2). The construct for mRNA microinjection does not contain the untranslated 5' sequence of zfPGRN-A. Hence there is no possibility of binding between mRNA and the morpholino when they are co-injected. Translation enhanced capped mRNA was synthesized with the mMessage mMachine Kit (Ambion, Huntingdon, England). For mRNA overexpression and rescue experiments 100 pg of either zfPGRN-A or hPGRN mRNA was used. Microinjection of a 1 ng of GFP mRNA was used as a control to demonstrate that the microinjection and over-expression does not inherently affect development. GFP/pcDNA3 vector was first transcribed and injected up to 1 ng per embryo and the GFP signal was observed with the enhanced GFP filter using a Leica MZFLIII stereomicroscope. The fluorescent GFP signal was observed uniformly throughout the body confirming that the mRNA was intact and translated into protein.

### Immunohistochemistry/immunofluorescence

Embryos (approximately 24 hpf) grown in egg water (water made from commercially available salt solution, 0.6% Instant Ocean) were supplemented with 0.003% phenylthiocarbamide (PTC) to prevent the appearance of melanin pigmentation. Staged embryos were manually dechorionated and fixed for 2 hours at room temperature or overnight at 4°C in 4% paraformaldehyde (PFA)/Phosphate Buffered saline (PBS). After several washes in PBS, embryos were stored in 100% methanol until required. Rehydration of embryos was performed for 5 min periods in successive solutions of Methanol/PBS with Tween (PBST) and then 3 times in PBST. Embryos were permeabilized with proteinase K diluted in PBST at a final concentration of 10 μg/ml. Post fixation was then carried out in 4% PFA/PBS for 20 min at room temperature followed by 3 rinses in PBST. Embryos were incubated with blocking buffer (5% Calf Serum, 1% DMSO in PBST) for 3-5 hours. Embryos were then incubated with znp1 (ZIRC) monoclonal antibody (1:200), a marker for motor neurons (MNs), Spinal cord, retinal and hindbrain axons or zfPGRN-A polyclonal antibody raised against a synthetic peptide corresponding to residues 242 to 256 of zfPGRN-A (RAEWEDHKQKKPETQC; synthesized by solid-phase chemistry at the Sheldon Biotechnology Centre of McGill University with a carboxyl-terminal cysteine to facilitate conjugation) which was diluted 1:500 in blocking buffer. Incubations were carried out overnight at 4°C followed by six washes in PBST. Embryos incubated with anti-znp1 were then treated with Goat anti-Mouse AP conjugate (Calbiochem) secondary antibody diluted to 1:1000 with blocking buffer in PBST for 2 hr at room temperature. Embryos incubated with anti-zfPGRN-A were then incubated with Alexa488 conjugated anti-rabbit antibody diluted to 1:1000 with blocking buffer in PBST for 2 hr at room temperature. Embryos incubated with both the secondary antibodies were washed six times in PBST. Embryos destined to be used to visualize znp1 labelling were incubated in staining buffer (100 mM Tris-HCl pH 9.5, 50 mM MgCl_2, _100 mM NaCl, 0.1% Tween-20,1 mM levamisol) and colour developed with NBT and BCIP-T (Fermentas). After 30 min the staining reaction was stopped and Caudal primary motor neurons (Cap MNs) within the trunk (excluding the tail region) of the embryos were visualized under an Olympus inverted phase contrast microscope. Pictures were taken with an Olympus DP12 camera and processed with Adobe Photoshop 7.0 software. The zfPGRN-A immunofluorescence was visualized with a Leica MZ FLIII stereomicroscope equipped with a GFP filter, photographed and processed with Adobe Photoshop 7.0 software.

### Analysis of Caudal Primary Motoneurons (CaP MNs)

Caudal primary motor axons in whole-mounted 26-28 hpf embryos labelled with Znp1 monoclonal antibody were analyzed. Only the trunk CaP MNs (12 pairs) were scored [36]. In wild type embryos all of these had grown beyond the ventral edge of the notochord into the ventral somite at 24 hpf. Trunk hemisegments were scored as 'branched' when nerves were branched at or above the ventral edge of the notochord. This strategy was employed to exclude naturally occurring branching that is sometimes observed ventral to the notochord. Trunk hemisegments were also scored as 'truncated' when nerves did not grow beyond the horizontal myoseptum. When more than one znp1 immunolabeled axon fascicle exited the spinal cord this was scored as a multiple exit. Embryos were counted with respect to how many of the nerves of the 12 pairs in each demonstrated a particular defect. For each treatment at least three experiments were performed and at least 50 embryos were scored per determination. Values were expressed as mean ± standard error of the mean.

### Statistics

Statistical significance among experimental groups was determined by one-way analysis of variance (ANOVA), followed by the Newman-Keuls multiple comparison test. Error bars represent s.e.m. Calculation was performed using GraphPad software (GraphPad Software Inc., San Diego, CA).

### Western blot of Zebrafish Embryos

At 24 hpf embryos were manually de-yolked and frozen in liquid nitrogen then stored at -80C. Samples were thawed and 30 uL of 2× Laemmli buffer was added and extracts boiled for 5 minutes. Embryo extracts were resolved on 10% acrylamide gels as 2 embryo equivalents per lane and transferred to nitrocellulose membrane. The blots were incubated in PBST with 1:4000 anti-zfPGRN-A or 1:10000 anti-hPGRN (R&D System) or 1:1000 anti-GFP (Open Biosystems) for 1 hour followed by extensive washing. After incubating with an anti-rabbit or anti-goat IgG-horseradish peroxidase (HRP)-conjugated secondary antibody (diluted 1:4,000) at room temperature for 1 hour blots were visualized using enhanced chemiluminescence (GE Healthcare) according to the manufacturer's instructions. Equivalence of protein loading per lane was determined by staining with mouse monoclonal β-actin antibody (AC-40; Sigma) at a dilution of 1:1000 followed by 1:4000 anti-mouse IgG-horseradish peroxidase (HRP)-conjugated secondary antibody. The rabbit polyclonal anti-zfPGRN-A was raised against synthetic peptide corresponding to residues 242 to 256 of zfPGRN-A (RAEWEDHKQKKPETQC), and residues 153 to 173 of zebrafish progranulin-B (CGSSPFLRKFAARRRKPLEKNA), respectively (Sheldon Biotechnology Centre). Epitope-specific immunoglobulin was purified by affinity chromatography. The specificity of western blot immunoreactive bands were confirmed by blocking incubations with synthetic zfPGRN-A and zfPGRN-B peptides.

## Competing interests

The research reported in this manuscript was supported in part work was supported by Neurodyn Inc. and HPJB is a member of the Scientific Advisory board of Neurodyn.

## Authors' contributions

BPC designed and conducted all the experiments, analyzed the data and wrote the manuscript. DCB participated in the preliminary studies with morpholino knockdowns and in the design of the study. DGK participated in the design of the study. AB and HPJB co-ordinated the design of the study and the writing of the manuscript. HPJB conceived of the study. All authors read and approved the final manuscript.

## Supplementary Material

Additional file 1 ***In vivo *translation of 1 ng gfp mRNA**. (A) bright field image showing no developmental abnormalities and (B) widespread appearance of gfp signal within the developing embryo at 27 hpf. Click here for file

Additional file 2 **Supplementary Table S1: Summary of the survival and swimming behaviour of embryos shown in videos 1-11 (Additional Files **3, 4, 5, 6, 7, 8, 9, 10, 11, 12, 13). Click here for file

Additional file 3 **Video 1: Touch-evoked swimming behaviour of wild-type embryo at 48 hpf**. Click here for file

Additional file 4 **Video 2: Reduced motility of zfPGRN-A deficient embryos to swim away when compared to wild-type embryos at 48 hpf**. Click here for file

Additional file 5 **Video 3: Touch-evoked swimming behaviour of wild-type embryo at 72 hpf**. Click here for file

Additional file 6 **Video 4: Inability of zfPGRN-A deficient embryos to swim away when compared to wild-type embryos at 72 hpf**. Click here for file

Additional file 7 **Video 5: Embryos co-injected with zfPGRN-A mRNA together with PGRN-A MO show rescued motility (48 hpf)**. Click here for file

Additional file 8 **Video 6: Embryos co-injected with hPGRN mRNA together with PGRN-A MO show rescued motility (48 hpf)**. Click here for file

Additional file 9 **Video 7: Embryos over-expressing zfPGRN-A show a strong avoidance swimming behaviour (48 hpf)**. Click here for file

Additional file 10 **Video 8: Embryos over-expressing hPGRN show a strong avoidance swimming behaviour (48 hpf)**. Click here for file

Additional file 11 **Video 9: smn1 knockdown embryos do not show the strong avoidance swimming phenotype typical of wild-type larvae (48 hpf)**. Click here for file

Additional file 12 **Video 10: Smn1 knockdown embryos co-injected with zfPGRN-A mRNA show an improved avoidance swimming phenotype when compared to that resulting from knockdown of smn1 (48 hpf)**. Click here for file

Additional file 13 **Video 11: Smn1 knockdown embryos co-injected with hPGRN mRNA show an improved avoidance swimming phenotype when compared to that resulting from knockdown of smn1 (48 hpf)**. Click here for file
