# Supplementary figures and images for: Progranulin modulates zebrafish motoneuron development in vivo and rescues truncation defects associated with knockdown of Survival motor neuron 1
Source: Mol Neurodegener. 2010 Oct 14;5:41. doi: 10.1186/1750-1326-5-41 (PMC2974670; doi:10.1186/1750-1326-5-41)

# 24hpf-gfp mRNA

A

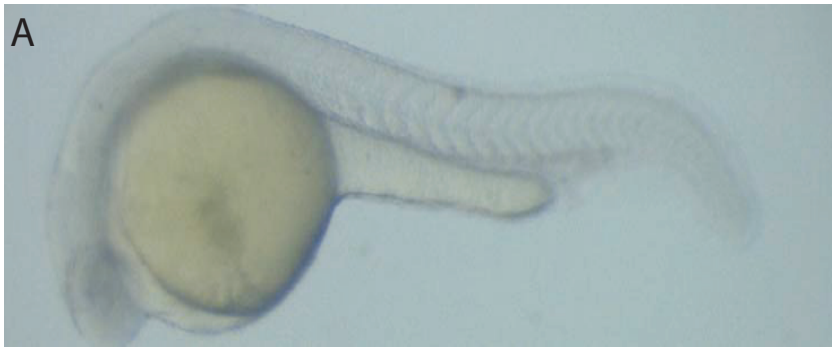

B

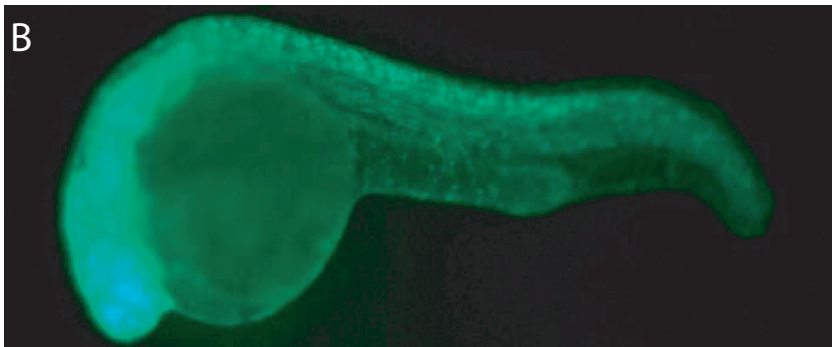

Supplement: Additional file 1 — In vivo translation of 1 ng gfp mRNA. (A) bright field image showing no developmental abnormalities and (B) widespread appearance of gfp signal within the developing embryo at 27 hpf. [file 1750-1326-5-41-S1.PDF]
