# Supplementary material for: Progranulin modulates zebrafish motoneuron development in vivo and rescues truncation defects associated with knockdown of Survival motor neuron 1
Source: Mol Neurodegener. 2010 Oct 14;5:41. doi: 10.1186/1750-1326-5-41 (PMC2974670; doi:10.1186/1750-1326-5-41)
Supplement: Additional file 2 — Supplementary Table S1: Summary of the survival and swimming behaviour of embryos shown in videos 1-11 (Additional Files 3, 4, 5, 6, 7, 8, 9, 10, 11, 12, 13). [file 1750-1326-5-41-S2.DOC]

| **Category** | **% survival (Day1)** | **% motor defect (Day2)** |
| --- | --- | --- |
| WT (n=30) | 97 | 7 |
| zfPGRN-A knockdown (n=50) | 72 | 69 |
| zfPGRN-A MO+zfPGRN-A mRNA(n=54) | 82 | 32 |
| zfPGRN-A MO+hPGRN-A mRNA (n=36) | 80 | 42 |
| zfPGRN-A mRNA (n=39) | 87 | 12 |
| hPGRN mRNA (n=27) | 85 | 17 |
| Smn1 knockdown (n=30) | 50 | 40 |
| Smn1 MO + zfPGRN-A mRNA (n=46) | 87 | 22 |
| Smn1 MO + hPGRN mRNA (n=59) | 91 | 18 |
